# Supplementary material for: A Qualitative Study on the Design and Implementation of the National Action Plan on Antimicrobial Resistance in the Philippines
Source: Antibiotics (Basel). 2022 Jun 17;11(6):820. doi: 10.3390/antibiotics11060820 (PMC9220349; doi:10.3390/antibiotics11060820)
Supplement: Supplementary file 1 [file antibiotics-11-00820-s001.zip › antibiotics-1687168-supplementary.pdf]

Appendix A: Question guide

SEMI-STRUCTURED QUESTION GUIDE FOR IN-DEPTH INTERVIEWS

<u>**Introduction**</u>

1. To begin, please share with us your professional experience over the last ten years. How long have you worked on AMR?

2. What is your opinion of AMR in your country? What do you think are the key drivers of AMR?

3. What are your views on implementation of National Action Plan on Antimicrobial Resistance (NAP) in your country?

<u>**Framing of AMR**</u>

4. How is the issue of AMR framed by the policymakers (Healthcare, Development, Innovation etc)? What were the contextual factors that shaped AMR getting into the agenda?
   - Political
   - Economical
   - Social

<u>**Policy design of NAP**</u>

Strategic vision

5. How did AMR become a policy issue in your country? Was there a situation analysis performed to determine the prevalence and incidence of AMR organisms?

6. What are the key action plans stipulated in NAP?
   - Education
   - Surveillance and risk assessment
   - Research
   - Prevention and control of infection
   - Optimisation of antimicrobial use

7. What was your role in the design of the NAP?

8. Are NAP objectives being measured through SMART indicators?

Participation and Coordination

9. Who were the other key actors involved in the design of the NAP?
   - Policy-makers and planners
   - Public, private and non-governmental sectors
   - Educational organizations and professional societies
   - Animal and human health sectors
   - Prescribers, practitioners and patients
   - Pharmacists and dispensers
   - Pharmaceutical industry
   - Civil society representatives
   - Aid agencies

10. How are the meetings organized? Is there coordination among the sectors?
    - What are the major challenges faced?

11. What are the key issues that discussed during these meetings? Are there any challenging discussions?

12. Overall, do you feel it was a participatory process? Were all actors involved equally?
    - If not, do you feel one actor was more involved or powerful in shaping this process?
    - Why do you think they are more powerful? What did they push for with their power?

13. Who do you think are the most important actors in driving the NAP policy formulation and implementation?

Appendix A: Question guide

Equity

14. How is the government standardizing regulation on antimicrobial drugs while ensuring that patients have access to these lifesaving medicines?

15. Do you think improving access to quality and affordable healthcare can reduce misuse of antibiotics?
    - What measures have been taken to achieve that?

Accountability and transparency

16. Please describe the process of being accountable (with regards to the NAP) to the government.
    - Is there a responsible committee, or is there a representative from each sector?
    - Are there plans in place in the event objectives are not met?
    - Are the progress reports publicly available?

Sustainability and funding

17. How do you think the sustainability of the NAP is ensured?
    - Dedicated budgets from appropriate forecast
    - Ongoing support from technical workgroup

18. Which areas have been prioritized for funding? Are the funds directed equally towards each arm of action, such as surveillance, research stewardship, infection prevention and control, and health promotion?
    - Why do you think certain areas have been prioritized over the other?
    - Is it evidence informed?

<u>**Implementation phase**</u>

19. It has been x years since the NAP was published in your country. How do you think the issue with AMR has changed over the past few years?
    - What are the main areas that require attention?

20. Are NAP objectives aligned with those of Global Action Plan on AMR?

21. Please share with us, which sectors (human/animal/plant health, food production/safety, and environment, public vs private) are actively involved in implementing the NAP over the past few years?
    - What are the roles played by each key actor involved (as discussed earlier) in implementing the NAP?

Here we will focus on participants' specific expertise which can be one or two of the areas listed below.

Education and public awareness

22. Please describe some of the existing strategies for improving awareness and understanding of AMR.
    - Are the messages tailored to the target audience?
        - Healthcare professionals - Are there measures taken to ensure the quality of materials used in training?
        - General public
        - Industry (farmers, wholesalers)

23. Are these AMR awareness campaigns/activities being measured?

24. Has your country invested in developing a health promotion campaign on sanitation, hygiene and vaccination? Is it still running?

25. What are the priority areas? Which areas require further action?

26. Please share with us the measures taken to educate and raise awareness about AMR in the animal health sector.

AMR Surveillance system and risk assessment

27. Please describe the existing surveillance system to combat AMR in your institution.
    - Which areas are done well? Which areas require further action?

28. Is there a national surveillance system that collects data across the sectors (human, animal, food production/safety, environment)?
    - How integrated is the national surveillance system?
    - Is there an existing guideline for national human AMR surveillance?
    - Is it being implemented well?
    - Who is accountable for AMR surveillance in your country?

29. Do you think there is sufficient laboratory capacity to monitor AMR?
    - How many labs have been identified so far?
    - Was quality assessment conducted for these labs?

30. Is information on national laboratory network publicly accessible?

31. How closely are the changes in antimicrobial exposure monitored? Is there an early warning system in place?

32. What are the important data that should be collected?
    - System designed for surveillance of antimicrobial use, that includes monitoring national level sales or consumption of antimicrobials in human/animal health services
        - Prescribing practices and quality of antimicrobials use monitored at a national sample of healthcare settings
        - Total sales of antimicrobials monitored at national and at sub-national level
    - Incidence/prevalence of resistant organisms

Research and innovation

33. What programmes are there to strengthen the knowledge and evidence base through better laboratory capacity and research opportunities?
    - *How do these programmes contribute towards a collective research agenda against AMR?*
    - *Is cross sector collaboration present?*

34. What are the plans for research & development and facilitating market access to novel antimicrobials, diagnostics, vaccines in both human and animal health?

35. Is there a dedicated national budget for AMR research?

Prevention and control of infection

36. What strategies are there to prevent and reduce of incidence of infection? How are these strategies being carried out on the ground?
    - Immunisation policies
    - Infection prevention and control guidelines/measures
    - Biosecurity in animal sector
    - Hygiene and sanitation in environmental sector

37. How does the country prevent contamination of the environment with antimicrobials?
    - Any existence of legislation or regulations for this?

38. Do you think adequate measures have been taken to prevent and reduce healthcare associated infections (HAI) in hospitals?
    - Is it applicable to both public and private healthcare setting?
    - Is data on HAI regularly reported?

Antimicrobial stewardship programme

39. What do you think of antibiotic stewardship as an infectious disease prevention strategy?
    - Have standard guidelines and protocol been made available to all hospitals?
    - Efforts taken to improve technical capacity of the staff implementing the stewardship programmes?

Medicine regulation for rational use of antimicrobials and surveillance of sale

40. Please share with me some of the strategies used to promote rational use of medicines.
    - Clinical practice guidelines and essential medicines list
    - Drug therapeutic committees
    - Provision of medicines information + continued professional development/education
    - Reduce financial incentives that encourage irrational use
    - Reduce antimicrobial use in food-producing animals

41. Would you like to share about the national policy that prevents over the counter sale of antibiotics in your country?
    - Is the ban on over the counter sale limited to specific antibiotics?
    - How regularly are the sales data from pharmacies reported?
    - Please share about the challenges faced by the regulatory authorities.
    - Does the regulatory policy extend to sale of antibiotics in the veterinary sector?

42. Is there an existing mechanism to monitor active pharmaceutical ingredients of antibiotics produced?
    - If so, please share about the regulatory policy.
    - Does it also regulate the import and export of approved antibiotics?

43. What impacts do economic and regulatory strategies have on changing prescribers'/farmers behaviour and AMR?
    - How does cost control policies impact on inappropriate antimicrobial prescribing?
    - How about enforcement of existing laws and regulations?

44. Let's discuss about over-prescription of antimicrobials. How does the prospect of litigation lead to defensive over-prescription of antimicrobials and increase occurrence and spread of AMR?

45. What are the effects of reimbursement, patient charges, and health insurance on AMR? For example:
    - Do these have an impact on inappropriate antimicrobial use?
    - To what extent does patient's economic situation lead to misuse of antimicrobials?

One Health engagement

46. Tackling AMR requires multi-sectoral response from all the sectors including human, animal and environment health. So far we have discussed about policies and gaps in human and animal health. Do you think adequate measures have been taken to regulate the release of antibiotic residue in the environment?
    - *Who is accountable?*
    - *What are the major challenges faced?*
    - *Educational activities?*
    - *Is there an existing protocol that mandates pharmaceutical companies and hospitals to manage the residue before its release?*
    - *What about the antibiotics residue that is released through faeces of food producing animals?*

Monitoring and evaluation

Reporting and feedback mechanism

48. Are there up-to-date progress/surveillance reports published regularly?
    - *Are they being feedback to the relevant governmental bodies? Why or why not?*
    - *Are they publicly available? Why or why not?*

49. Does the leading institution set regular deadlines for the implementing organisations to review the progress made?

Effectiveness

50. Are technical experts consulted when implementation challenges are flagged by the organisations?

51. Have there been efforts to increase competency and technical capacity of the implementing staff?

52. How effective do you think the action plan is thus far?
    - *Have there been efforts to evaluate the effectiveness of the NAP? (Overall and specific strategies)*

53. Now that we have discussed the NAP at the national level, is there anything else that you would like to add with regards to what your institution is doing for the NAP?

54. What is your forecast for AMR scenario (regionally and internationally) based on the current policy and interventions in place?
    - *Where are the gaps?*
    - *How can the resources be spent better?*

We have come to the end of our discussion. Thank you for participating in this session and I really appreciate your time spent here today. Do you have anything more to add to this discussion or have any questions to ask?

Appendix B: COREQ checklist

COREQ (COnsolidated criteria for REporting Qualitative research) Checklist

A checklist of items that should be included in reports of qualitative research. You must report the page number in your manuscriptwhere you consider each of the items listed in this checklist. If you have not included this information, either revise your manuscriptaccordingly before submitting or note N/A.

Topic	Item No.	Guide Questions/Description	Reported on Page No.
**Domain 1: Research team and reflexivity**			
Personal characteristics			
Interviewer/facilitator	1	Which author/s conducted the interview or focus group?	4
Credentials	2	What were the researcher's credentials? E.g. PhD, MD	N/A
Occupation	3	What was their occupation at the time of the study?	N/A
Gender	4	Was the researcher male or female?	N/A
Experience and training	5	What experience or training did the researcher have?	4
Relationship with participants			
Relationship established	6	Was a relationship established prior to study commencement?	4
Participant knowledge of the interviewer	7	What did the participants know about the researcher? e.g. personal goals, reasons for doing the research	4
Interviewer characteristics	8	What characteristics were reported about the inter viewer/facilitator? e.g. Bias, assumptions, reasons and interests in the research topic	N/A
**Domain 2: Study design**			
Theoretical framework			
Methodological orientation and Theory	9	What methodological orientation was stated to underpin the study? e.g. grounded theory, discourse analysis, ethnography, phenomenology, content analysis	4
Participant selection			
Sampling	10	How were participants selected? e.g. purposive, convenience, consecutive, snowball	3
Method of approach	11	How were participants approached? e.g. face-to-face, telephone, mail, email	3
Sample size	12	How many participants were in the study?	3
Non-participation	13	How many people refused to participate or dropped out? Reasons?	3
Setting			
Setting of data collection	14	Where was the data collected? e.g. home, clinic, workplace	4
Presence of non-participants	15	Was anyone else present besides the participants and researchers?	4
Description of sample	16	What are the important characteristics of the sample? e.g. demographic data, date	3
Data collection			
Interview guide	17	Were questions, prompts, guides provided by the authors? Was it pilot tested?	4
Repeat interviews	18	Were repeat interviews carried out? If yes, how many?	4
Audio/visual recording	19	Did the research use audio or visual recording to collect the data?	4
Field notes	20	Were field notes made during and/or after the interview or focus group?	4
Duration	21	What was the duration of the interviews or focus group?	4
Data saturation	22	Was data saturation discussed?	4
Transcripts returned	23	Were transcripts returned to participants for comment and/or correction?	N/A

Appendix B: COREQ checklist

Topic	Item No.	Guide Questions/Description	Reported on Page No.
**Domain 3: analysis and findings**			
Data analysis			
Number of data coders	24	How many data coders coded the data?	4
Description of the coding tree	25	Did authors provide a description of the coding tree?	4
Derivation of themes	26	Were themes identified in advance or derived from the data?	4
Software	27	What software, if applicable, was used to manage the data?	4
Participant checking	28	Did participants provide feedback on the findings?	N/A
Reporting			
Quotations presented	29	Were participant quotations presented to illustrate the themes/findings? Was each quotation identified? e.g. participant number	5-13
Data and findings consistent	30	Was there consistency between the data presented and the findings?	5-13
Clarity of major themes	31	Were major themes clearly presented in the findings?	5-13
Clarity of minor themes	32	Is there a description of diverse cases or discussion of minor themes?	5-13

Developed from: Tong A, Sainsbury P, Craig J. Consolidated criteria for reporting qualitative research (COREQ): a 32-item checklist for interviews and focus groups. International Journal for Quality in Health Care. 2007. Volume 19, Number 6: pp. 349 – 357

Once you have completed this checklist, please save a copy and upload it as part of your submission. DO NOT include this checklist as part of the main manuscript document. It must be uploaded as a separate file.
